# Prevalence of post-intensive care syndrome among Japanese intensive care unit patients: a prospective, multicenter, observational J-PICS study

**DOI:** 10.1186/s13054-021-03501-z

**Published:** 2021-02-16

**Authors:** Daisuke Kawakami, Shigeki Fujitani, Takeshi Morimoto, Hisashi Dote, Mumon Takita, Akihiro Takaba, Masaaki Hino, Michitaka Nakamura, Hiromasa Irie, Tomohiro Adachi, Mami Shibata, Jun Kataoka, Akira Korenaga, Tomoya Yamashita, Tomoya Okazaki, Masatoshi Okumura, Takefumi Tsunemitsu

**Affiliations:** 1 Department of Anesthesia and Critical Care, Kobe City Medical Center General Hospital, 2-1-1, Minatojima minamimachi, Chuo-ku, Kobe-City, Hyogo Prefecture 650-0047 Japan; 2 Department of Emergency and Critical Care Medicine, St. Marianna University School of Medicine, Kawasaki, Kanagawa Prefecture 216-8511 Japan; 3 Department of Clinical Epidemiology, Hyogo College of Medicine, Nishinomiya, Hyogo Prefecture 663-8501 Japan; 4 Department of Emergency and Critical Care Medicine, Seirei Hamamatsu General Hospital, Hamamatsu, Shizuoka Prefecture 430-8558 Japan; 5 Department of Emergency and Critical Care Medicine, Hiroshima General Hospital, Hatsukaichi, JAHisoshima Prefecture 738-8503 Japan; 6 Emergency and Critical Care Center, Kurashiki Central Hospital, Kurashiki, Okayama Prefecture 710-8602 Japan; 7 Department of Critical Care Medicine, Nara Prefecture General Medical Center, Nara, Nara Prefecture 630-8581 Japan; 8 Department of Anesthesiology, Kurashiki Central Hospital, Kurashiki, Okayama Prefecture 710-8602 Japan; 9 Emergency and Critical Care Center, Tokyo Women’s Medical University Medical Center East, Tokyo, 116-8567 Japan; 10 Department of Emergency and Critical Care Medicine, Wakayama Medical University, Wakayama, Wakayama Prefecture 641-8510 Japan; 11 Department of Critical Care Medicine, Tokyo Bay Urayasu Ichikawa Medical Center, Urayasu, Chiba 279-0001 Japan; 12 Department of Emergency Medicine, Japanese Red Cross Wakayama Medical Center, Wakayama, Wakayama Prefecture 640-8558 Japan; 13 Department of Emergency and Critical Care, Osaka City General Hospital, Osaka, 534-0021 Japan; 14 Emergency Medical Center, Kagawa University Hospital, Kita, Kagawa Prefecture 761-0793 Japan; 15 Department of Anesthesiology, Aichi Medical University Hospital, Nagakute, Aichi Prefecture 480-1195 Japan; 16 Department of Emergency Medicine, Hyogo Prefectural Amagasaki General Medical Center, Hyogo Prefecture, Amagasaki, 660-8550 Japan

**Keywords:** Post-intensive care syndrome, Health-related quality of life, SF-36, Disability, Critical care, Mechanical ventilation, Intensive care unit

## Abstract

**Background:**

Many studies have compared quality of life of post-intensive care syndrome (PICS) patients with age-matched population-based controls. Many studies on PICS used the 36-item Short Form (SF-36) health survey questionnaire version 2, but lack the data for SF-36 values before and after intensive care unit (ICU) admission. Thus, clinically important changes in the parameters of SF-36 are unknown. Therefore, we determined the frequency of co-occurrence of PICS impairments at 6 months after ICU admission. We also evaluated the changes in SF-36 subscales and interpreted the patients’ subjective significance of impairment.

**Methods:**

A prospective, multicenter, observational cohort study was conducted in 16 ICUs across 14 hospitals in Japan. Adult ICU patients expected to receive mechanical ventilation for > 48 h were enrolled, and their 6-month outcome was assessed using the questionnaires. PICS definition was based on the physical status, indicated by the change in SF-36 physical component score (PCS) ≥ 10 points; mental status, indicated by the change in SF-36 mental component score (MCS) ≥ 10 points; and cognitive function, indicated by the worsening of Short-Memory Questionnaire (SMQ) score and SMQ score at 6 months < 40. Multivariate logistic regression model was used to identify the factors associated with PICS occurrence. The patients’ subjective significance of physical and mental symptoms was assessed using the 7-scale Global Assessment Rating to evaluate minimal clinically important difference (MCID).

**Results:**

Among 192 patients, 48 (25%) died at 6 months. Among the survivors at 6 months, 96 patients responded to the questionnaire; ≥ 1 PICS impairment occurred in 61 (63.5%) patients, and ≥ 2 occurred in 17 (17.8%) patients. Physical, mental, and cognitive impairments occurred in 32.3%, 14.6% and 37.5% patients, respectively. Population with only mandatory education was associated with PICS occurrence (odds ratio: 4.0, 95% CI 1.1–18.8, *P* = 0.029). The MCID of PCS and MCS scores was 6.5 and 8.0, respectively.

**Conclusions:**

Among the survivors who received mechanical ventilation, 64% had PICS at 6 months; co-occurrence of PICS impairments occurred in 20%. PICS was associated with population with only mandatory education. Future studies elucidating the MCID of SF-36 scores among ICU patients and standardizing the PICS definition are required.

*Trial registration* UMIN000034072.
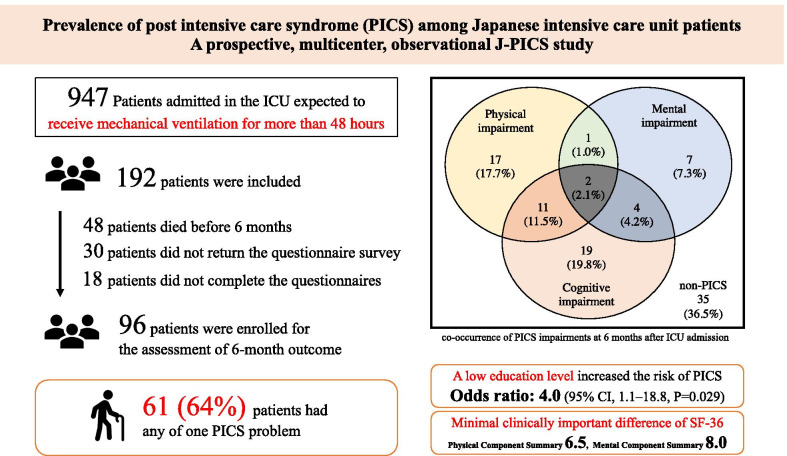

**Supplementary Information:**

The online version contains supplementary material available at 10.1186/s13054-021-03501-z.

## Background

Recent improvement in intensive care unit (ICU) mortality rate has resulted in numerous ICU survivors. Most ICU survivors experience long-term impairment in their quality of life, known as post-intensive care syndrome (PICS). PICS is defined as a new or worsening impairment in physical, cognitive or mental health status arising and persisting after hospitalization for critical illness [1]. Globally, many observational studies have been conducted for each PICS parameter among ICU patients [2–5]. These studies have several limitations. First, most studies focused on only one or two aspects of PICS (i.e., only cognitive function, or only physical and mental status). Only a few studies focusing on the co-occurrence of PICS symptoms in individual patients have been conducted [6, 7]. Second, limited data regarding PICS after implementation of PICS prophylaxis strategy are available, especially for Asian populations with different cultural backgrounds and races from Western countries [8–11]. Many guidelines and bundle strategies aimed at growing awareness about PICS have been published and implemented to decrease the occurrence of PICS. These include clinical practice guidelines for the prevention and management of pain, agitation, sedation, delirium, immobility and sleep disruption in adult patients in the ICU [12]; guidelines for family-centered care in the neonatal, pediatric and adult ICU [13]; and ABCDEFGH bundle [14]. ABCDEFGH bundle comprises airway management; breathing trials; coordination of care and communication; delirium assessment; early mobility; family involvement, follow-up referrals, and functional reconciliation; good handoff communication; and handout materials on PICS and PICS family. The Japanese PICS survey conducted in 2019 revealed that more than 60% medical staff recognized PICS, and early rehabilitation was provided in more than 90% cases [15]. Third, ICU patients often report lower health-related quality of life (HRQOL) than the general population before ICU admission. However, many studies have compared quality of life of PICS patients with age-matched population-based controls, instead of baseline quality of life [16]. Furthermore, many studies on PICS used the 36-item Short Form (SF-36) health survey questionnaire version 2, which is the most popular HRQOL scale for the outcome measure, among ICU survivors [17]; however, these studies lack the data for SF-36 values before and after ICU admission. Thus, the clinically important changes in the parameters of SF-36 in these studies are unknown.

Therefore, the Japanese PICS (J-PICS) study aimed to evaluate the co-occurrence of PICS symptoms, along with the assessment of baseline HRQOL, in Japanese patients admitted to multicenter ICUs, after publication of several guidelines and bundles about PICS prophylaxis. Additionally, we evaluated the subjective significance of changes in SF-36 parameters before and after ICU admission among the patients.

## Methods

### Study design and setting

The J-PICS study was a prospective, multicenter, observational cohort study of mechanically ventilated patients, conducted in 16 ICUs across 14 hospitals of Japan. Five of the 16 ICUs were university-affiliated hospitals, and the others were tertiary teaching hospitals. The median number of ICU beds was 10 [interquartile range (IQR), 8–15]. All ICUs were mixed ICUs and were managed by the intensivists. The study protocol was approved by the Kobe City Medical Center General Hospital (KCGH) ethics committee and the ethics committees of all participating hospitals (KCGH approval number: Zn181008). Written informed consent was obtained from all patients or authorized surrogates. The J-PICS study has been registered at University Hospital Medical Information Network Clinical Trials Registry (registration number: UMIN000034072).

### Study population and eligibility criteria

All consecutive adult ICU patients who were expected to receive mechanical ventilation for more than 48 h between April 01, 2019, and September 30, 2019, were recruited in the study. The patients who received noninvasive mechanical ventilation were also enrolled. Eligibility criteria were assessed next morning at 8:00 am after admission in the ICUs. The patients with the following conditions were excluded from the study: (1) patients who had primary brain injury that was likely to result in conscious or cognitive disorder (e.g., traumatic brain injury, subarachnoid hemorrhage, acute stroke, post cardiac arrest, meningitis, and encephalitis); (2) patients with pre-admission diagnosis of dementia; (3) patients who received home ventilation prior to admission; (4) patients with end-stage cancer; (5) patients with withdraw/withhold status; (6) expected death within 24 h; (7) second or subsequent admission to ICU during the study period; (8) patients who had no family members; (9) patients who did not speak Japanese; and (10) patients who could not be followed-up (e.g., did not live in Japan and/or were homeless).

### Variables and measurements

The following demographic and hospital data of the patients were collected: age, sex, body mass index, Charlson comorbidity index (CCI) [18], clinical frailty scale [19], do not attempt resuscitation code status at the time of ICU admission, educational level, employment status, marital status, patient’s residential living status before admission, history of treatment with benzodiazepines and steroids, source of admission to ICU, and primary diagnosis at the time of ICU admission. We chose 9 years as the cutoff value for educational level because elementary and junior high school is mandatory for everyone, which usually last for 9 years in Japan, following which, many people go to high school for three years, followed by universities and graduate schools. The number of patients with sepsis and acute respiratory distress syndrome (ARDS) was also recorded. Sepsis and ARDS were diagnosed on the basis of Sepsis-3 [20] and Berlin definition [21], respectively. The severity of illness was measured using the Acute Physiology and Chronic Health Evaluation (APACHE II) score and Sequential Organ Failure Assessment (SOFA) score [22] within 24 h of ICU admission. The management data in the ICU, including the use of inotropes or vasopressors, paralytic agents except during intubation, renal replacement therapy, extracorporeal membrane oxygenation (ECMO), intra-aortic balloon pump and tracheostomy, were also collected. The data for the use of inotropes, vasopressors and paralytic agents were collected during the first four days of ICU admission. The data on patient outcomes, including ICU mortality; in-hospital mortality; length of stay in ICU; length of stay in hospital; duration of mechanical ventilation; occurrence of delirium, diagnosed by the Confusion Assessment Method for the Intensive Care Unit [23], during the first four days; and discharge status among survivors were collected.

### Patient-reported outcomes

Six months after ICU admission, the authors sent the questionnaires by post to all patients except those who died. Patient-reported data were collected centrally. If the participants did not revert, the lead author asked each participating institution to attempt a contact via telephone to return the questionnaire.

The patient-reported outcome survey evaluated the physical and mental functions of patients through the assessment of HRQOL and cognitive functions. HRQOL was assessed using the SF-36 questionnaire [24–26], which is available in the Japanese language [27, 28]. At the time of enrollment in the study after ICU admission, the patients’ baseline SF-36 questionnaire was completed by a proxy (4-week recall assessment before the patients’ current acute illness). At 6 months after ICU admission, SF-36 questionnaire was obtained by mail from the patient or proxy. The SF-36 questionnaire has established acceptability, reliability, and validity in critically ill patient populations and as a surrogate-completed proxy measure [29–36]. SF-36 questionnaire is a comprehensive 36-item survey of HRQOL with two summary scales, physical component scale (PCS) and mental component scale (MCS), with scores ranging from 0 to 100. A higher score indicates better physical and mental functions. Both scales were transformed to a normalized scale using norm-based scoring (NBS) with 50 as the population mean and 10 points representing one standard deviation. The scores were calculated based on the standard methods [37]. The missing data were treated using the standard methods. If a patient answered more than half of the items on the subscale, the missing data were replaced with the mean of the subscale. In contrast, if a patient answered less than half items on the subscale, the data of the answered questions were excluded [37].

Cognitive functioning was assessed using the Short-Memory Questionnaire (SMQ) [38]. The SMQ was to be completed by a proxy, similar to the Informant Questionnaire on Cognitive Decline in the Elderly [39]. The SMQ is the only questionnaire filled by a proxy that has been translated and established in the Japanese language [40]. The SMQ is a 12-item questionnaire with scores ranging from 4 to 46. A score less than 40 indicates cognitive dysfunction.

The primary outcome was the occurrence of PICS 6 months after ICU admission. The definition of PICS was any of the following criteria: (1) decline in physical status, indicated by PCS score decrease of ≥ 10 points; (2) decline in mental status, indicated by MCS score decrease of ≥ 10 points; or (3) cognitive function impairment, indicated by decline in SMQ score and SMQ score < 40 at 6 months after ICU admission. A 10-point change was considered clinically important in a previous study in the ICU setting [36]. For the assessment of subjective significance of SF-36 score change, participants also answered anchor questions on whether their physical and mental status had improved, worsened or remained unchanged on a 7-point Likert scale (from large negative change to large positive change) [41–43] at 6 months. The questions related to patients’ employment status at 6 months were also asked.

### Statistical analyses

Since the analyses focused on patient-reported outcomes at 6 months, participants who were lost to follow-up and who died before 6 months were excluded from the final analysis. Sample size was based on the total number of patients meeting the inclusion criteria because this prospective study was descriptive and hypothesis generating by nature. Continuous variables are presented as medians and interquartile range. Categorical variables are presented as absolute values and percentages. A univariate analysis was performed using the Wilcoxon rank-sum test for continuous variables, and the Chi-squared test or Fisher’s exact test was used for categorical variables, if the number was less than 10. Multivariate logistic regression model was used to identify the factors associated with the occurrence of PICS. Multivariate analysis included the following variables as confounding factors: age, APACHE II score, CCI and educational status [7, 44–46]. Age, APACHE II score and CCI were log-transformed. The risk of PICS is expressed as odds ratio (OR) with 95% confidence interval (CI). The number of missing data have been reported, and no assumptions have been made except for SF-36 questionnaire data. The changes in SF-36 PCS, MCS and SMQ scores between baseline and 6 months after ICU admission were compared using the Wilcoxon signed-rank test.

The subjective significance of change in SF-36 PCS and MCS was assessed by an anchor-based question with a Global Assessment Rating as large negative change, moderate negative change, small negative change, no change, small positive change, moderate positive change and large positive change in physical and mental status. The change in each Global Assessment Rating for SF-36 PCS and MCS scores is expressed as mean and standard deviation (SD). Further, we evaluated the responsiveness of SF-36 PCS and MCS. Responsiveness is the instrument’s ability to detect change over time in the construct to be measured [47, 48]. Responsiveness of SF-36 PCS and MCS scores to negative change or no change in Global Assessment Rating at 6 months was calculated using the effect size index [49, 50]. Cohen’s d effect size was used to evaluate the changes as small (0.2–0.49), moderate (0.5–0.79) and large (> 0.80) [47, 51]. We also assessed interpretability, which is the degree to which one can assign qualitative meaning to the quantitative scores or change in scores. We also evaluated the floor effect and ceiling effect of SF-36 PCS and MCS scores. Floor and ceiling effects were calculated as the percentage of participants scoring their lowest and highest (0 and 100) score, respectively. The floor and ceiling effects were considered relevant at 15% [52]. The minimal clinically important difference (MCID) of SF-36 PCS and MCS scores was calculated as the mean change score for no change patients minus the mean change score for small negative change patients [53, 54].

The differences with *P* value less than 0.05 (two-sided) were considered statistically significant. All data were analyzed using JMP 15.1.0 (SAS Institute Inc., Cary, NC, USA).

## Results

### Patients’ characteristics

**Fig. 1:**
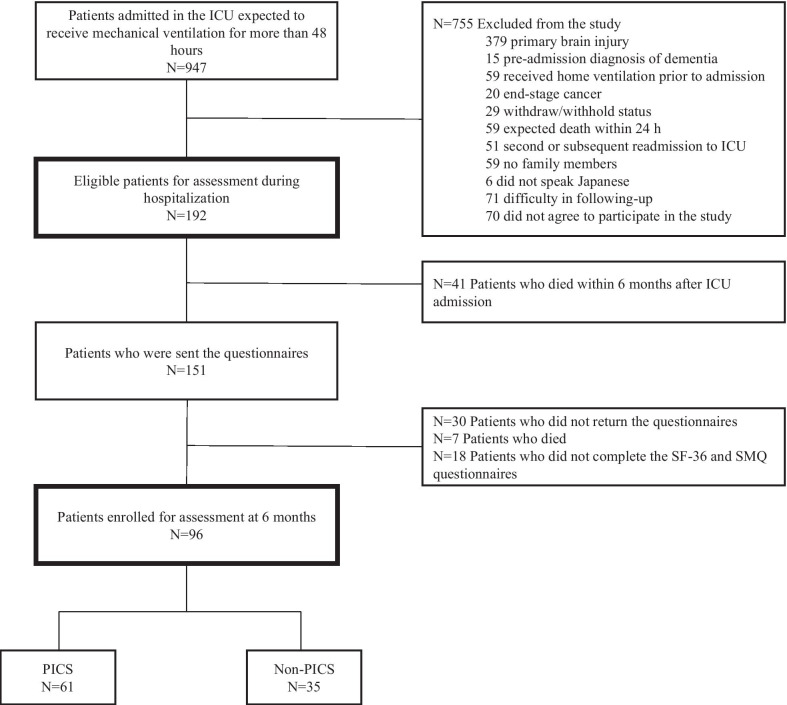
Flowchart depicting the enrollment of subjects in the study. *ICU* Intensive care unit, *SF-36* 36-item short-form health survey questionnaire, *SMQ* Short-Memory Questionnaire, *PICS* post-intensive care syndrome

During the study period, 947 consecutive patients were registered in the J-PICS study, among whom, 755 patients were excluded from the study based on the exclusion criteria. Finally, 192 patients were included in the study. Among 192 patients, 30 (15.6%) patients did not return the questionnaire survey, 48 (25.0%) patients died before 6 months of follow-up, and 18 (9.4%) patients did not complete the questionnaires. Thus, in total, 96 (50.0%) patients were enrolled for the assessment of 6-month outcome (Fig. 1).

Overall characteristics of the patients, along with stratification based on the presence and absence of PICS, are shown in Table 1. In total, 61 (63.5%) patients were in the PICS group and 35 (36.5%) in the non-PICS group. The mean age of the patients was 74 [64–81] years, the proportion of men was 65%, mean APACHE II score was 23 [18–28], and mean SOFA score was 8 [6–11]. The main cause of ICU admission was acute respiratory failure. There were 35% and 41% patients with ARDS and sepsis, respectively. Population with only mandatory education was associated with PICS occurrence.

**Table 1:** Patients’ characteristics: overall and stratified by the presence and absence of PICS

	non-PICS (N = 35)	PICS (N = 61)	*P* value	Overall (N = 192)
Age, years	75 [64–79]	74 [59–81.5]	0.77	74 [64–81]
Male, N (%)	25 (71.4)	43 (70.5)	0.92	125 (65.1)
BMI, kg/m^2^	21.9 [19.5–24.4]	22.8 [19.7–25.3]^a^	0.21	21.9 [19.5–25.0]^b^
APACHE II score	21 [18–25]	20 [14.5–25]	0.14	23 [18–28]
SOFA score	7 [4–10]	8 [5–10]	0.21	8 [6–11]
Charlson comorbidity index	1 [0–2]	1 [0–2]	0.9	1 [0–3]
Clinical frailty scale	3 [2–4]^a^	3 [2–4]	0.67	3 [3, 4]^a^
DNAR status at ICU admission, N (%)	1 (2.9)	2 (3.3)	1	15 (7.8)
Educational level, N (%)	N = 33*	N = 60*	0.098	N = 177
≤ 9 years	3 (9.1)	15 (25)		49 (27.7)
> 9 years	30 (91.0)	45 (75)		128 (72.3)
Employment status, N (%)	N = 34*	N = 61*	0.16	N = 188
Student	0 (0)	0 (0)		2 (1.1)
Employed or self-employed	11 (32.4)	23 (37.7)		60 (31.9)
Unemployed	3 (8.8)	11 (18)		41 (21.8)
Housework	2 (5.9)	8 (13.1)		21 (11.2)
Retired	18 (52.9)	19 (31.2)		64 (34.0)
Marital status, N (%)			0.64	
Married	25 (71.4)	39 (63.9)		119 62.0)
Separated or divorced	1 (2.9)	6 (9.8)		16 (8.3)
Widowed	5 (14.3)	9 (15.8)		37 (19.3)
Unmarried	4 (11.4)	7 (11.5)		20 (10.4)
Patient's residential living status, N (%)			0.25	
Lived alone at home	3 (8.6)	12 (19.7)		33 (17.2)
Lived with someone else	32 (91.4)	48 (78.7)		152 (79.2)
Nursing home	0 (0)	1 (1.6)		7 (3.6)
History of treatment with benzodiazepines, N (%)	3 (8.6)	7 (11.5)	0.74	18 (9.4)
History of treatment with steroids, N (%)	2 (5.7)	4 (6.6)	1	35 (18.2)
Source of admission to ICU, N (%)			0.19	
Emergency department	20 (57.1)	23 (37.7)		95 (49.5)
Hospital floor	5 (14.3)	16 (26.2)		45 (23.4)
Another hospital	1 (2.9)	0 (0)		3 (1.6)
Operating room (elective)	0 (0)	1 (1.6)		4 (2.1)
Operating room (emergency)	9 (25.7)	21 (34.4)		45 (23.4)
Primary diagnosis at the time of admission in ICU, N (%)			0.075	
Cardiogenic	2 (5.7)	13 (21.3)		25 (13.0)
Acute respiratory failure	18 (51.4)	16 (26.2)		67 (34.9)
Infection	8 (22.9)	13 (21.3)		56 (29.2)
Trauma	3 (8.6)	8 (13.1)		16 (8.3)
Others	4 (11.4)	11 (18)		28 (14.6)
ARDS, N (%)	5 (14.3)	12 (19.7)	0.51	35 (18.2)
Sepsis, N (%)	11 (31.4)	20 (32.8)	0.89	79 (41.1)
Management in ICU, N (%)				
Inotrope/vasopressor	27 (77.1)	50 (82.0)	0.6	148 (77.1)
Paralytic agent	3 (8.6)	9 (14.8)	0.53	24 (12.5)
Renal replacement therapy in ICU	6 (17.1)	6 (9.8)	0.34	40 (20.8)
ECMO in ICU	0 (0)	4 (6.6)	0.29	7 (3.6)
IABP in ICU	0 (0)	3 (4.9)	0.3	6 (3.1)
Tracheostomy	4 (11.4)	9 (14.8)	0.76	29 (15.1)

Data are presented as median [interquartile range] or number (percentage)

*PICS* post-intensive care syndrome, *BMI* body mass index, *APACHE II* Acute Physiology and Chronic Health Evaluation, *SOFA* Sequential Organ Failure Assessment, *DNAR* do not attempt resuscitation, *ICU* intensive care unit, *ARDS* acute respiratory distress syndrome, *ECMO* extracorporeal membrane oxygenation, *IABP* intra-aortic balloon pump

^a^One missing datum

^b^Three missing data

*The education status and the employment status of the remaining persons were unknown

### Hospital outcomes

Among the patients included in the study, ICU mortality, hospital mortality and 6-month mortality were observed in 15 (7.8%), 33 (17.2%) and 48 (29.6%) patients, respectively. Table 2 shows the hospital outcomes and 6-month outcomes among the patients who were enrolled for assessment at 6 months after ICU admission. The median ICU length of stay, hospital length of stay and duration of mechanical ventilation were 7 [5–14] days, 33.5 [19–61.8] days and 6 [3–11] days, respectively. Among 159 survivors, discharge destination was home, another facility and nursing home for 71 (44.7%), 87 (54.7%) and 1 (0.7%) patient, respectively. Among non-PICS patients, 22 (62.9%) were discharged home, while among PICS patients, 24 (39.3%) were discharged home from the hospital (*P* = 0.026).

**Table 2:** Patients’ hospital and 6-months outcome: overall and stratified by the PICS status

	non-PICS (N = 35)	PICS (N = 61)	*P* value	Overall (N = 192)
Mortality				
ICU mortality	NA	NA		15 (7.8)
Hospital mortality	NA	NA		33 (17.2)
6-Month mortality	NA	NA		48 (29.6)^a^
ICU length of stay (days)	7 [5–12]	7 [5–13]	0.78	7 [5–14]
Hospital length of stay (days)	28 [17–47]	39 [19–71.5]	0.10	33.5 [19–61.8]
Days of mechanical ventilation	5 [3–7]	5 [3–11]	0.96	6 [3–11]
Delirium, N (%)	11 (31.4)	20 (32.8)	0.89	61 (31.8)
Discharged from hospital among survivors, N (%)			0.026	N = 159
Home	22 (62.9)	24 (39.3)		71 (44.7)
Another facility	13 (37.1)	37 (60.1)		87 (54.7)
Nursing home	0	0		1 (0.7)

Data are presented as median [interquartile range] or number (percentage)

*PICS* post-intensive care syndrome, *ICU* intensive care unit, *NA* not applicable

^a^30 missing data

### Patient-reported outcomes at 6 months

#### SF-36 score

**Fig. 2:**
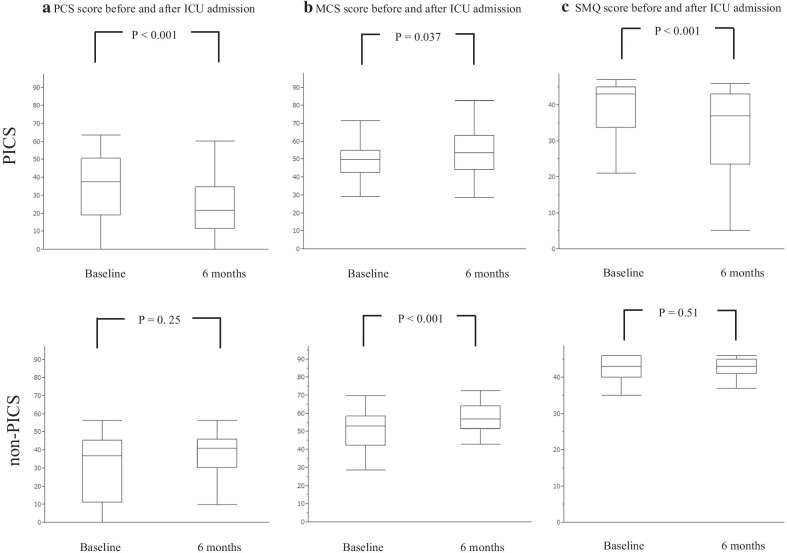
Changes in SF-36 PCS, MCS and SMQ scores before and after ICU admission. *SF-36* 36-item short-form health survey questionnaire, *PCS* physical component scale, *MCS* mental component scale, *SMQ* Short-Memory Questionnaire, *ICU* intensive care unit

Figure 2 shows that SF-36 PCS score and MCS score changed significantly at 6 months compared with the baseline among both PICS and non-PICS patients. Among PICS patients, PCS score at baseline and 6 months was 37.6 [18.9–50.8] and 21.5 [11.4–34.8], respectively (*P* < 0.001), and MCS score at baseline and 6 months was 49.7 [42.5–55.0] and 53.4 [44.2–63.4], respectively (*P* = 0.037). In contrast, among non-PICS patients, PCS score at baseline and 6 months was 36.7 [11.2–45.4] and 41.0 [30.2–46.0], respectively (*P* = 0.25), and MCS score at baseline and 6 months was 53.0 [42.2–58.6] and 56.8 [51.6–64.2], respectively (*P* < 0.001). Physical and mental impairment, defined by the worsening of PCS and MCS scores by more than 10 points, occurred in 31 (32.3%) and 14 (14.6%) patients, respectively.

#### SMQ score

Figure 2 also shows the change in SMQ score from baseline to 6 months among both PICS and non-PICS patients. Among PICS patients, SMQ score at baseline and 6 months was 43 [33.8–45] and 37 [23.5–43], respectively (*P* < 0.001), while it was 43 [40–46] and 43 [41–45], respectively (*P* = 0.51), in non-PICS patients. Cognitive impairment, defined by the worsening of SMQ score and SMQ score at 6 months < 40, occurred in 36 (37.5%) patients.

#### PICS

**Fig. 3:**
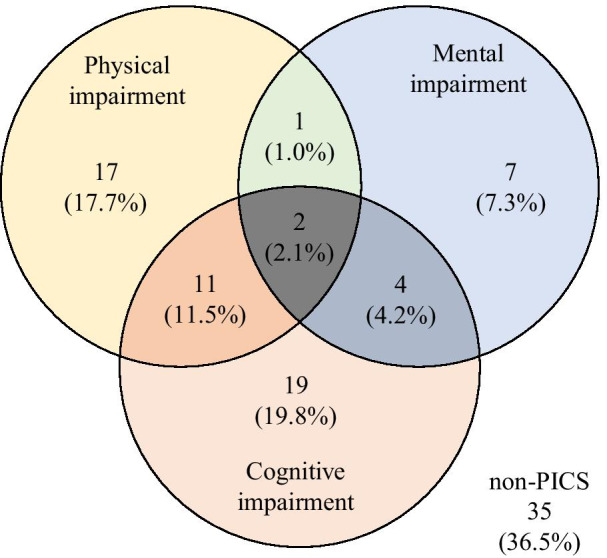
Occurrence of PICS problems among patients at 6 months after ICU admission. *PICS* post-intensive care syndrome

In total, 96 patients completed all surveys about physical, mental and cognitive status. The number of patients whose baseline PCS and MCS scores were < 50, and SMQ score was < 40 and was 74 (77.1%), 46 (47.9%) and 30 (31.3%), respectively. PICS was observed in 61 (63.5%) patients. The proportion of patients with one, two and three PICS impairments is shown in Fig. 3. Table 3 shows the results of multivariate analysis. After adjusting for confounding variables, population with only mandatory education (OR: 4.0, 95% CI 1.1–18.8, *P* = 0.029) was an independent predictor for the occurrence of PICS at 6 months after ICU admission. When education level was analyzed as a continuous variable, the same result was obtained (*P* = 0.009).

**Table 3:** Multivariate logistic regression analysis for PICS

	OR (95% CI)	*P* value
Ln (Age)	0.33 (0.036–2.4)	0.28
Ln (APACHE II score)	0.31 (0.067–1.1)	0.081
Ln (Charlson comorbidity index + 1)*	1.3 (0.62–2.8)	0.28
Population with mandatory 9-year education	4.0 (1.1–18.8)	0.029

*PICS* post-intensive care syndrome, *OR* odds ratio, *CI* confidence interval, *APACHE* Acute Physiology and Chronic Health Evaluation

*1 was added to Charlson comorbidity index before performing log transformation because the data had the values of 0

#### Return to work

Among patients who completed the 6-month outcome survey, the data of four patients regarding job were missing. Among the remaining patients, 31 (33.7%) patients (20 in PICS group and 11 in non-PICS group) worked prior to ICU admission. The number of patients who returned to work was 26 (83.9%). All patients of non-PICS group returned to work, while 15 (75%) patients of PICS group returned to work.

#### Subjective significance of SF-36 score

The subjective significance of change in SF-36 PCS and MCS scores assessed by Global Assessment Rating was not obtained in 3 patients. Additional file 1: Table 1 shows the change in SF-36 PCS and MCS scores based on change in Global Assessment Rating. The effect size index between Global Assessment Rating negative change and change in PCS and MCS scores was 0.72 and 0.50, respectively. Area under receiver operating characteristics curve between Global Assessment Rating negative change and change in PCS and MCS scores was 0.69 and 0.64, respectively. These values indicate moderate responsiveness between the change in SF-36 PCS and MCS scores and negative change in Global Assessment Rating. Floor effect of PCS score at baseline and 6 months was 4.3% and 3.2%, respectively. Ceiling effect of PCS score and floor and ceiling effect of MCS score were not observed. MCID of PCS and MCS scores was 6.5 and 8.0, respectively.

## Discussion

### Summary of key findings

In total, 192 ICU patients who were expected to receive mechanical ventilation for more than 48 h were registered in the J-PICS study. The 6-months mortality rate was 30%. The prevalence of PICS at 6 months after ICU admission was 64% among 96 adult ICU survivors. New or worsened physical, mental and cognitive impairments occurred in 32.3%, 14.6% and 37.5% patients, respectively. The percentage of patients who had two or more PICS impairments was 17.8%. Population with only mandatory education was associated with the occurrence of PICS. MCID of PCS and MCS scores was 6.5 and 8.0, respectively, as analyzed by anchor-based subjective questions.

### Comparison to other studies

This study investigated the co-occurrence of PICS symptoms at 6 months after ICU admission. To the best of our knowledge, only two studies have investigated the co-occurrence of PICS symptoms. One was a small cohort study, wherein the study population comprised patients with ICU length of stay of at least 2 days [6]. This study reported the occurrence of PICS among the patients, and at least one PICS impairment was high in 84% of the patients. The prevalence of two or more PICS impairments was 56%. Another study [7], which comprised the patients with respiratory failure or shock, reported that the prevalence of PICS was 64% at 3 months and 56% at 12 months after hospital discharge. Two or more PICS impairments occurred in 25% patients at 3 months and 21% patients at 12 months. The results of the latter study were similar to those of our cohort. In contrast, the results of the former study showed a higher prevalence of PICS than the results of our cohort. This difference may be due to the difference in baseline data. In our cohort, baseline SF-36 PCS and MCS scores were lower than NBS. The former study did not evaluate the baseline status, and the latter study excluded patients with preexisting physical, mental and cognitive impairments. Additionally, the former study reported that PICS occurred in 54% of the survivors and these patients reported worse symptoms after critical illness. The aforementioned observations suggest that the evaluation of baseline status is an important factor for interpreting the prevalence of PICS. The exclusion of patients with preexisting physical, mental and cognitive impairments was also effective in interpreting the prevalence of PICS. However, that leads to the exclusion of many patients from the study. Therefore, we should evaluate the baseline status of the patients in PICS study.

In the present study, we found significant difference in discharge destination of the patients between the PICS and non-PICS groups. Many patients in the PICS group were transferred to other hospitals where the duration of treatment was associated with the severity of underlying illness. Thus, there is a possibility that hospital length of stay, including that in another hospital, might have affected the PICS symptoms. Therefore, several confounding factors might have affected our results.

In our study, population with only mandatory education was an independent predictor for the occurrence of PICS. This observation is consistent with that of the previous studies, which demonstrated that population with only mandatory education was associated with physical impairment [49], mental impairment [55] and PICS [7]. Socioeconomic position is a broad term which assesses the socioeconomic status, including education level, occupation and income. Underprivileged is associated with poor health due to behavioral risk factors. The underprivileged persons often have high rates of cardiovascular disease and mortality due to tobacco smoking and less physical activity [55]. Furthermore, those who received less formal education is associated with low income, small social network to use resources for recovery, less coping ability for the rehabilitation process and less accurate recall [7, 49, 55, 56]. Additionally, financial distress has a direct effect on mental status [57].

In the present study, 84% patients returned to work at 6 months after ICU admission. The proportion of patients returning to work in this study was higher than that reported in the previous study [58, 59]. The patients in our study were older and lower proportion of persons worked before ICU admission as compared to previous studies [58, 59]. One ARDS longitudinal follow-up study in Canada reported that return to work rates at 1 year and 5 years were 63% (40 of 64 previously employed survivors) and 92% (49 of 53 survivors), respectively [58]. Another ARDS study conducted in the USA reported return to work rates at 1 year and 5 years as 49% (28 of 67 previously employed survivors) and 44% (28 of 64 survivors), respectively [59]. These differences were potentially explained by the differences in patient population (e.g., age and diagnosis at admission), and socioeconomic or geographic factors [59]. It has also been reported that return to work is driven by economic necessity and difficulties in obtaining administrative financial support [60].

We also evaluated the subjective significance of SF-36 score among ICU patients. We evaluated the responsiveness and MCID of only negative change in Global Assessment Rating because the patients with positive change were less in number and our focus was to analyze PICS, i.e., a negative change. This study showed moderate responsiveness between the change in SF-36 PCS and MCS scores and negative change in Global Assessment Rating. A floor effect was observed, but no ceiling effect. Clinically significant differences, rather than statistically significant differences, are important to assess HRQOL. The smallest meaningful change in a score is indicated by MCID. In the present study, we used a 10-point change in PCS or MCS score as the criterion of PICS. However, some studies have used a 5-point change as the criterion of PICS [61]. In the present study, MCID of PCS and MCS scores was 6.5 and 8.0, respectively, as analyzed by anchor-based subjective questions. A 5-point change might have overestimated PICS. MCID can be determined by two approaches: anchor-based method and distribution method. MCID values should be determined by systematic review and evaluation processes such as the modified Delphi method [62]. Our study had a small sample size, and the evaluation was done only by an anchor-based method. Future study with a larger population is required to clarify the MCID of SF-36 scores among ICU patients to standardize the definition of PICS globally.

### Strengths and limitations

The present study has several strengths. First, we investigated not only the PICS symptoms but also the co-occurrence of PICS symptoms. Second, we assessed the baseline status of the patients. Third, we evaluated the MCID of SF-36 score change. Fourth, the J-PICS study was the first multicenter observational study of PICS in Japan. To date, a few studies have investigated the occurrence of PICS in Asian populations.

The present study also has some limitations. First, the long-term outcomes were assessed at only one time point. However, a systematic review showed that 60% of the previous studies about PICS have reported a single follow-up assessment. Furthermore, there were 71% articles without baseline assessment of quality of life [63]. We did not do multiple follow-up assessments, but we incorporated baseline assessment in our study. The systematic review about trajectory of recovery after ICU showed that HRQOL, cognitive function and return to work always rapidly improved during the first 6 months, whereas improved a little after 1 year [64–66]. For this reason, a single follow-up at 6 months is appropriate for assessing the PICS symptoms.

Second, the definition of PICS has not gained a consensus yet. In 425 articles about PICS, 250 different measurement instruments were used [63]. Furthermore, we evaluated the clinimetric property of SF-36. The previous studies assessed all three domains originally defined for PICS using cutoff for each measurement instrument [6, 7]. Recently, expert consensus for screening of postdischarge impairments was published [67]. However, research agenda about measurement instruments based on follow-up strategies, like in-person interview (in clinic or online), telephone interview or post questionnaires, was not decided. Third, our data may have some bias; for example, recall bias because the outcome measures were self-reported by the patients or proxies. Fourth, we could not include a large number of patients in our study, which is also the limitation of majority of the similar studies. This might have affected the mortality rate and prevalence of PICS.

## Conclusions

Our data suggest that 64% of ICU survivors had PICS impairments. Approximately 20% of survivors had a co-occurrence of PICS impairments. The less literate was associated with the occurrence of PICS. Future studies are needed to elucidate the modifiable risk factors of PICS to prevent the occurrence of PICS. We attempted to clarify the MCID of SF-36 among the ICU population. MCID of PCS and MCS scores was 6.5 and 8.0, respectively. More research is required for the assessment of MCID of SF-36 among the ICU population. Additionally, generalizability of the definition of PICS is needed.

## Supplementary Information

**Additional file 1: Table 1**. Changes in SF-36 PCS and MCS scores based on 7-point Global Assessment Rating

## Data Availability

The datasets used and analyzed during the current study are available from the corresponding author on reasonable request.

## Abbreviations

APACHE II: Acute Physiology and Chronic Health Evaluation II
ARDS: Acute respiratory distress syndrome
BMI: Body mass index
CI: Confidence interval
CCI: Charlson comorbidity index
DNAR: Do not attempt resuscitation
ECMO: Extracorporeal membrane oxygenation
HRQOL: Health-related quality of life
IABP: Intra-aortic balloon pump
ICU: Intensive care unit
IQR: Interquartile range
KCGH: Kobe City Medical Center General Hospital
MCID: Minimal clinically important difference
MCS: Mental component scale
NBS: Norm-based scoring
OR: Odds ratio
PCS: Physical component scale
PICS: Post-intensive care syndrome
RRT: Renal replacement therapy
SD: Standard deviation
SF-36: 36-Item Short Form
SMQ: Short-Memory Questionnaire
SOFA: Sequential Organ Failure Assessment
