# Supplementary material for: Prevalence of post-intensive care syndrome among Japanese intensive care unit patients: a prospective, multicenter, observational J-PICS study
Source: Crit Care. 2021 Feb 16;25:69. doi: 10.1186/s13054-021-03501-z (PMC7888178; doi:10.1186/s13054-021-03501-z)
Supplement: Supplementary file 1 — Additional file 1: Table 1. Changes in SF-36 PCS and MCS scores based on 7-point Global Assessment Rating [file 13054_2021_3501_MOESM1_ESM.docx]

**Additional Table 1. Changes in SF-36 PCS and MCS scores based on a 7-point Global Assessment Rating**

|  | Large negative change | Moderate negative change | Small negative change | No change | Small positive change | Moderate positive change | Large positive change |
| --- | --- | --- | --- | --- | --- | --- | --- |
|  | N=17 | N=15 | N=29 | N=18 | N=8 | N=4 | N=2 |
| Change in SF-36 PCS score | -19.2 (23.4) | -11.5 (19.7) | -1.4 (15.8) | 5.1 (15.0) | 2.3 (17.7) | 4.9 (15.6) | 8.9 (6.7) |
|  | N=6 | N=12 | N=22 | N=39 | N=7 | N=5 | N=2 |
| Change in SF-36 MCS score | -2.6 (17.3) | 5.3 (14.2) | -0.8 (13.6) | 7.2 (10.9) | 4.4 (11.8) | 11.9 (12.9) | 17.5 (3.0) |

Data are presented as mean (standard deviation).

**Abbreviations:** SF-36: 36-item Short Form health survey questionnaire, PCS: Physical component scale, MCS: Mental component scale
